# Al(SO_4_)(OH)·5H_2_O Stemming from Complexation of Aluminum Sulfate with Water-Soluble Ternary Copolymer and further Stabilized by Silica Gel as Effective Admixtures for Enhanced Mortar Cementing

**DOI:** 10.3390/ma17194762

**Published:** 2024-09-27

**Authors:** Zhiyuan Song, Zainab Bibi, Sidra Chaudhary, Qinxiang Jia, Xiaoyong Li, Yang Sun

**Affiliations:** 1 Department of Applied Chemistry, School of Chemistry, Xi’an Jiaotong University, No. 28, Xianning West Road, Xi’an 710049, China; 13038012682@163.com (Z.S.); zainabbibi55@stu.xjtu.edu.cn (Z.B.); sidra-ch576@stu.xjtu.edu.cn (S.C.); qinxiangjia1984@mail.xjtu.edu.cn (Q.J.); lixy6658@xjtu.edu.cn (X.L.); 2 Shanxi Jiawei New Material Co., Ltd., Taijia Village, Jiedian Town, Wanrong County, Yuncheng 044200, China; 3 Xi’an Biomass Green Catalysis and Advanced Valorization International Science and Technology Cooperation Base, No. 28, Xianning West Road, Xi’an 710049, China

**Keywords:** ternary copolymer, aluminum sulfate, cement, mortar, setting time

## Abstract

A water-soluble ternary copolymer bearing carboxyl, sulfonic, and amide functional groups was synthesized using ammonium persulfate-catalyzed free radical polymerization in water, resulting in high monomer conversion. This copolymer was then complexed with aluminum sulfate, forming an admixture containing Al(SO_4_)(OH)·5H_2_O, which was subsequently combined with silica gel. Characterization revealed that the synthesized copolymer formed a large, thin membrane that covered both the aluminum compounds and the silica gel blocks. The introduction of this complex admixture, combining the copolymer and aluminum sulfate, not only reduced the setting times of the cement paste but also enhanced the mechanical strengths of the mortar compared to using aluminum sulfate alone. The complex admixture led to the formation of katoite, metajennite, and C_3_A (tricalcium aluminate) in the mortar, demonstrating significant linking effects, whereas pure aluminum sulfate could not completely transform C_3_S within 24 h. Further addition of silica gel to the complex admixture further shortened the setting times of the paste, slightly reduced compressive strength, but improved flexural strength compared to the initial complex admixture. The silicon components appeared to fill the micropores and mesopores of the mortar, accelerating cement setting and enhancing flexural strength, while slightly decreasing compressive strength. This study contributed to the development of new cementing accelerators with improved hardening properties.

## 1. Introduction

The rational design and application of organic polymers in cement engineering have garnered significant attention in both academic and industrial sectors [1]. Cement-based materials, such as concrete, exhibit excellent properties for construction, including high castability, good mortar fluidity, satisfactory compressive strength, impressive fire resistance, and low production costs [2]. These materials also face several critical drawbacks. These include poor flexural strength, high brittleness, and susceptibility to sulfate (acid) attack and chloride corrosion [2].

Ordinary Portland Cement (OPC) primarily consists of C_3_S (alite, 3CaO·SiO_2_), which produces C–S–H (3CaO·2SiO_2_·3H_2_O) upon hydration, a colloidal gel that binds various concrete components such as sand, limestone, chalk, and gypsum [3]. However, the formation of C–S–H tends to exhibit a flaky, two-dimensional morphology rather than a robust three-dimensional network, leading to numerous dislocations and defects [3]. This ultimately results in a reduced durability of concrete. Consequently, enhancing the intrinsic properties and external performance of C–S–H presents a significant challenge for both scientists and engineers.

According to the previous literature, the incorporation of polymers into the cementing process can significantly enhance the properties of cement-based concrete, thereby improving its applicability and suitability for specific construction purposes [4,5]. In practice, the most commonly used polymer additives can be categorized into two main types based on their primary functions: superplasticizers and functional filler agents.

Superplasticizers have emerged as some of the most widely utilized concrete admixtures in civil engineering, particularly for high-performance and self-compacting concretes. Their primary function is to reduce water content and porosity in cementitious materials by coating fine cement particles and preventing the agglomeration of cement clusters [4]. This enhancement significantly improves the mechanical strength and durability of the concrete mixture [4].

For example, when OPC is hydrated under high temperatures and pressures, such as in the cementing of gas and oil wells, there is a need for highly dispersed cement particles and slower hydration rates to achieve suitable thickening and setting times [5]. In such scenarios, polycarboxylate ether (PCE) has been developed as a highly effective superplasticizer. PCE works by generating negative charges at high pH levels from the hydration water, which then tightly adsorb onto the positively charged cement particles [6]. This action inhibits the formation and agglomeration of early hydration products, primarily affecting the early development of C–S–H gel [6].

While a small dosage of polycarboxylate ether (PCE) can achieve good concrete workability, its fluidity retention properties still require improvement. Most known PCE compounds struggle to maintain their original fluidity for more than three hours [4]. Conversely, the addition of retarders such as sucrose, phosphates, and sodium gluconate can help mitigate fluidity loss; however, excessive use of these retarders can lead to overly prolonged setting times, bleeding, and even significant strength loss [7].

Recent research has indicated that significant structural modifications using hydrolyzable groups—such as amides, sulfonic acids, or esters—can enhance fluidity without introducing excessive retarding effects [8,9]. In this context, a newly developed polyacrylate-based ether superplasticizer has demonstrated superior dispersibility and stability, along with exceptional fluidity retention. This improvement is primarily attributed to its functional groups and the corresponding core–shell nanomicellar configuration [4].

Another category of polymer admixtures used in mortar cementing is functional stuffing agents. For instance, poly (ethylene-*co*-vinyl acetate) (EVA) offers several advantages, including a glossy surface, clear framework, stress-crack and UV resistance, and excellent waterproof properties. Mortar incorporating EVA has demonstrated significantly improved flexibility, higher adhesion strength, enhanced performance at low temperatures, better plastic viscosity, and effective noise insulation [10,11,12]. Typically, the hydration reaction of cement leaves behind a substantial number of capillary pores in the mortar, which can weaken its inherent strength. However, the incorporation of EVA into the mortar fills these pores, resulting in increased flexural strength and overall durability [10,11].

The autogenous shrinkage is widely recognized as a significant challenge in cementitious materials, particularly in those with a low water-to-binder ratio. This type of shrinkage occurs in the absence of external or internal water sources, leading to cracking that jeopardizes the durability of structures and the safety of residents [13].

To mitigate the effects of external humidity, the incorporation of superabsorbent polymers (SAPs) in cementitious materials has proven effective in reducing autogenous shrinkage. When self-desiccation begins, these polymers release stored water, driven by capillary forces. Additionally, the use of SAPs can alter the rheology and microstructure of mortar, enhancing its freeze–thaw resistance [14,15,16]. In practical applications, copolymers of acrylamide and sodium acrylate, as well as cross-linked potassium salt polyacrylate, have both been demonstrated to be effective SAPs in mortar cement [13].

The advancements in admixture technology have significantly improved the performance of cement-based materials. A range of admixtures, including accelerators, retarders, repair agents, corrosion inhibitors, and heat-adsorbing agents, have been introduced to the cementing process. These admixtures are tailored to address specific conditions, such as high-alkaline environments, intense agitation, sulfate (acid) attack, or chloride corrosion. Their use enables the achievement of desired performance characteristics under varying circumstances.

However, it is crucial to preserve the integrity of these delicate admixtures within the cement until they are activated. Microencapsulation has emerged as an effective method for maintaining admixture stability. The choice of an appropriate wall material for fabricating microcapsules is critical for ensuring the survival and controlled release of functional admixtures [17]. Given the variety of preparation methods available, using organic polymers as wall materials is a practical approach for both protecting and releasing admixtures. In practice, wall materials such as methyl methacrylate (MMA) [18], toluene diisocyanate (TDI) [19], and dicyclopentadiene (DCPD) [20] have demonstrated effective coating results in mortar cementing applications.

In addition to polymer admixtures used in cementing, inorganic additives, commonly referred to as mineral admixtures, also play a crucial role in modulating cement setting. Well-known mineral admixtures include silica fume (SF, primarily composed of SiO_2_) [21], clays [22], fly ash (FA) [23], and rice husk ash (RHA) [24], each exhibiting distinct effects on concrete quality. Overall, the benefits of mineral admixtures can be attributed to their contribution to the hardening process of concrete. Additionally, these admixtures help regulate the setting time of cement-based materials by influencing factors such as water demand, heat of hydration, setting time, bleeding, and reactivity.

In particular, there is a growing interest in examining the setting effects of silica-containing compounds, driven by their abundant market availability and low cost. For instance, studies have shown that incorporating silica fume (SF) at a dosage of 8–10% by mass of cement can produce 50,000 to 100,000 microspheres per cement particle, resulting in significantly denser and more cohesive concrete [25]. Additionally, SF typically exhibits a very fine spherical morphology, which contributes to its high reactivity with Ca(OH)_2_ generated during cement hydration. This reaction is crucial for enhancing mechanical strength, as Ca(OH)_2_ crystals are relatively weak and brittle, possessing non-cementitious properties that make them prone to cracking [26]. By utilizing SF, the consumption of Ca(OH)_2_ can mitigate this issue, leading to improved concrete performance.

Furthermore, it has been observed that negatively charged silica particles can attract to positively charged aluminate phases, thereby suppressing the active dissolution of C_3_A (tricalcium aluminate, 3CaO·Al_2_O_3_) [27]. This interaction enhances the sulfate resistance of cementitious materials with high aluminate content by contributing to the filling effect and reducing the expansion associated with crystal size and morphology (e.g., by decreasing the length-to-diameter ratio of crystals) [27]. Clearly, there remains significant potential to explore more effective silica admixtures to further improve cement setting properties.

In practice, there is also a significant demand for reduced setting times and high early mechanical strength in cold climates or in specific construction scenarios, such as tunnels, bridges, and roadways. Without these properties, the integrity and longevity of such structures could be compromised [28]. In this context, sprayed concrete (shotcrete), which is applied under high pressure and at high velocity, has been selected as a suitable method. Accelerators, such as aluminum sulfate (AS, Al_2_(SO_4_)_3_·18H_2_O), play a crucial role as key admixtures to expedite the setting and solidification of the mortar [28].

From a mechanistic perspective, the hydration of cement gradually increases the pH level. Subsequently, Al^3+^ ions released from the dissolution of aluminum sulfate (AS) undergo hydrolysis to form [Al(OH)_4_]^−^. This species then reacts with Ca^2+^ ions to produce C_3_A [29]. In turn, C_3_A reacts with gypsum (CaSO_4_·2H_2_O) to form AFt (ettringite, calcium sulfoaluminate hydrate, 3CaO·Al_2_O_3_·3CaSO_4_·32H_2_O). The formation of AFt creates a viscous three-dimensional network that interlinks various concrete components, thereby enhancing the early strength development of the material [29].

However, the use of aluminum sulfate (AS) as a cementing accelerator presents several challenges. Firstly, the rapid hydrolysis of AS leads to the formation of a substantial amount of AFt, which coats the surface of C_3_S particles. This coating can obstruct the penetration of water into C_3_S, thereby inhibiting the formation of C–S–H [28]. Secondly, AS has relatively low solubility (36.5 g per 100 g of water at 20 °C) [30], necessitating a higher water content in the cement mixture. This increased water demand can adversely affect the long-term strength development of the mortar. Consequently, optimizing the use of AS remains an area requiring further research and refinement.

To achieve both accelerating and hardening effects in the cementing of Ordinary Portland Cement (OPC), a copolymer composed of acrylic acid (AA), 2-acrylamido-2-methyl propane sulfonic acid (AMPS), and aconitic acid (AcA) was synthesized through an (NH_4_)_2_S_2_O_8_-initiated aqueous free radical polymerization. This copolymer was then complexed with aluminum sulfate (AS) to serve as the primary admixture in the hydration process of OPC. The resulting complex was further stabilized using silica gel, yielding a derivative admixture. The study evaluated not only the setting times of the cement paste but also the mechanical properties of the mortar, with a thorough discussion on the types and dosages of the admixtures used. Additionally, both microstructural and morphological characterizations were conducted to elucidate the cementing mechanisms involved. This research contributes to the advancement of cementitious materials.

## 2. Experimental

### 2.1. Starting Materials

In the synthesis of the admixtures, the monomers used included AMPS (2-acrylamido-2-methylpropane sulfonic acid, 98%), AA (acrylic acid, 99%), and AcA (*cis*-aconitic acid, 98%). The initiator used was (NH_4_)_2_S_2_O_8_ (ammonium persulfate, 98%), and *n*-butanol (*n*-BuOH, 99%) was also employed. All of these chemicals were procured from Shanghai Macklin Biochemical Technology Co., Ltd. Additionally, the silica gel (SiO_2_, with a purity of 99% and a mesh size of 300) was sourced from Qingdao Haiyang Chemical Co., Ltd., Qingdao, China.

For the determination of monomer conversion in the synthesized copolymer, the following reagents were utilized: KBrO_3_ (potassium bromate, 99.5%), KBr (potassium bromide, 99%), HgSO_4_ (mercuric sulfate, 99%), concentrated H_2_SO_4_ (sulfuric acid, 98%), NaCl (sodium chloride, 99.5%), KI (potassium iodide, 99%), and Na_2_S_2_O_3_ (sodium thiosulfate, 99%). All chemicals were procured from Shanghai Aladdin Biochemical Technology Co., Ltd., Shanghai, China.

To measure the setting time of cement paste and the mechanical strength of mortar, AS (aluminum sulfate, Al_2_(SO_4_)_3_·18H_2_O, 99%) was obtained from Shanghai Aladdin Biochemical Technology Co. Ltd., Shanghai, China. Cement (P·O 42.5) was supplied by the China National Academy of Building Materials Science Co. Ltd. Chinese ISO standard sand, fabricated according to GB/T 17671 [31], was purchased from Xiamen ISO Standard Sand Co. Ltd., Xiamen, China. Distilled water was prepared in our laboratory.

### 2.2. Instruments for Measuring Setting Time of Cement Paste and Mechanical Strength of Mortar

Both the initial setting time (IST) and final setting time (FST) of the cement paste were measured using a Vicat apparatus after the cement and admixtures were combined and mixed in a NJ-160A cement paste mixer. The cement mortar was prepared in a JJ-5 cement mortar mixer. All three devices were manufactured by Wuxi Xiyi Building Material Instrument Factory (Wuxi City, China).

The compressive and flexural strengths of the mortar were measured using a fully automatic anti-folding and compression testing machine, WAY-300B, which features an automatic pressure testing machine control system, EHC-2300, with a maximum capacity of 300 kN and a pressing speed of 48 N s^−1^. The mortar was cured in a numerical control standard cement conservation box, HBY-40B, at 20 °C and 90% humidity. Both instruments were manufactured by Wuxi Xiyi Building Material Instrument Factory (Wuxi City, China).

### 2.3. Instruments for Microstructural and Morphological Characterizations

FT-IR spectra of the tested samples were recorded using potassium bromide pellets on a Bruker Tensor 27 spectrometer (Billerica, MA, USA). X-ray photoelectron spectroscopy (XPS) measurements were conducted with a Kratos Axis Ultra DLD (Kratos Co., Ltd., Manchester, UK), utilizing monochromatic Al-Kα X-rays (1486.6 eV) as the excitation source. The binding energy scale was calibrated using the C 1s peak at 284.8 eV (sp^3^ hybridized, saturated carbon) as the reference. Peak fitting was performed with a Gaussian–Lorentz (G/L) product function, applying a 30% Lorentzian ratio.

Wide-angle X-ray diffraction (XRD) patterns of the powdered samples were obtained using a Philips X’Pert Pro diffractometer (PANalytical B.V. Co., Ltd., Almelo, The Netherlands), employing Cu-Kα radiation (λ = 1.5418 Å) with a scan rate of 0.05° s^−1^. Thermogravimetric analysis, including both TGA and DTG, of the prepared samples was conducted on a NETZSCH TG 209C with a TASC 414/4 controller under nitrogen protection (made by NETZSCH, Selby, Germany), utilizing a heating rate of 10 °C min^−1^ over a temperature range of 25–800 °C. Scanning electron microscopy (SEM) was performed using a Zeiss Sigma 300 (made by Zeiss, Oberkochen, Germany). Transmission electron microscopy (TEM) was performed on JEOL JEM-200CX at 120 kV (made by JEOL, Tokyo, Japan). Inductively Coupled Plasma Optical Emission Spectroscopy (ICP-OES) was carried out on an Agilent 5110 (made by Agilent Technologies, Santa Clara, CA, USA), with a pump rate of 60 rpm, plasma gas flow of 12.0 L min^−1^, nebulizer flow of 0.70 L min^−1^, stable time of 20 s, auxiliary gas flow of 1.0 L min^−1^, reading access time of 5 s, and sample flush time of 20 s, with RF power set at 1250 W.

### 2.4. Synthesis of Admixtures

**Figure 1:**
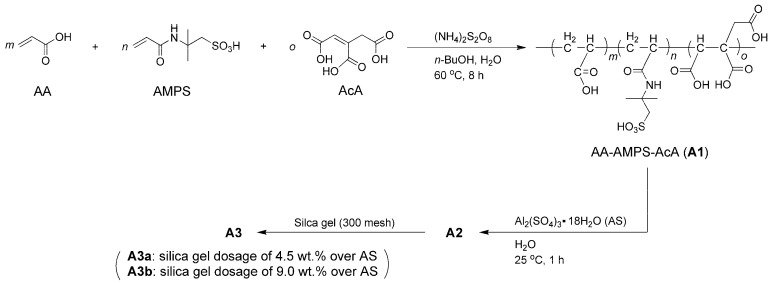
Synthesis of admixtures.

As illustrated in Figure 1, AA (100 g, 1.38 mol), AMPS (100 g, 0.48 mol), AcA (100 g, 0.57 mol), and *n*-BuOH (200 mL, 162.96 g, 2.19 mol) were combined with distilled H_2_O (400 mL) in a 1 L three-necked flask equipped with a condenser, addition funnel, and magnetic stirrer. A solution of (NH_4_)_2_S_2_O_8_ (2 g, 8.6 mmol, dissolved in 100 mL distilled H_2_O) was added slowly through the addition funnel under vigorous stirring at 25 °C over the course of 1 h. The temperature was then gradually increased to 60 °C while maintaining continuous stirring, and the mixture was stirred at this temperature for an additional 8 h. After cooling to room temperature, the solvent was completely removed via rotary evaporation, yielding a slightly yellow oil designated as *p*(AA–*co*–AMPS–*co*–AcA) (A1).

Next, A1 (100 g) and AS (350 g) were mixed with distilled water (150 g) in a 1 L three-necked flask equipped with mechanical stirring at 25 °C. After vigorous stirring for 1 h at 25 °C, the resulting white emulsion was decanted and stored for future use, designated as A2. Additionally, A2 (600 g) was combined with silica gel (15.75 g and 31.5 g, respectively) in a 1 L round-bottomed flask equipped with a mechanical stirrer at 25 °C. After vigorous stirring for 1 h at 25 °C, the resulting mixtures were obtained as A3a and A3b, respectively.

### 2.5. Determination of Monomer Conversion of Copolymer (A1)

The bromine number (*X*, mg g⁻^1^), representing the bromine consumption per gram of sample, was determined according to the Chinese standard GB/T 10535-2014 [32]. In principle, Br_2_ (generated in situ) covalently adds to the unpolymerized monomers present in the tested sample. Excess Br_2_ subsequently reacts with the added KI solution, and the precipitated I_2_ is quantified through standard titration with Na_2_S_2_O_3_.

In practice, A1 (0.5000 g) was placed in a 250 mL volumetric flask, and distilled H_2_O was added to reach a fixed volume. A 25.00 mL aliquot of this solution was then transferred to a 250 mL iodine flask (brown flask), followed by the addition of a mixed solution of KBrO_3_ and KBr (10 mL), prepared by dissolving 5.5 g of KBrO_3_ and 20.0 g of KBr in distilled H₂O to a total volume of 1000 mL in a brown volumetric flask. After shaking the mixture for 5 min, 20 mL of a 3 mol L^−1^ H_2_SO_4_ solution was added, followed by 5 mL of a HgSO_4_ solution prepared by dissolving 15 g of HgSO_4_ in 14 mL of concentrated H_2_SO_4_ and diluting it to 475 mL with distilled H_2_O. The resulting solution was thoroughly mixed and then stored in a dark place for 30 min at 0–20 °C.

Next, 15 mL of NaCl solution (116 g L^−1^) and 10 mL of KI solution (100 g L^−1^) were added, and the mixture was shaken well and stored in a bright place for 5 min at room temperature. Subsequently, 20 mL of distilled H_2_O was added. The solution was then titrated with a standard Na_2_S_2_O_3_ solution (0.1 mol L^−1^) until a slight yellow color appeared. After that, 1 mL of starch indicator solution (10 g L^−1^) was introduced, and titration continued until the solution changed from blue to colorless.

The bromine number (*X*, mg g^−1^) was calculated using Equation (1):

(1) $$X=\frac{c\times (V_{0}-V)\times 0.0799\times 1000}{m}$$

where *X*—bromine number (mg g^−1^); *c*—real concentration of standard Na_2_S_2_O_3_ solution (mol L^−1^); *V*_0_—consumed volume of standard Na_2_S_2_O_3_ solution in sample blank experiment; *V*—consumed volume of standard Na_2_S_2_O_3_ solution in regular experiment; 0.0799—the bromine (Br) mass (g) derived from the consumption of ideal Na_2_S_2_O_3_ solution (1 mL, 1.000 mol L^−1^); *m*—mass of tested sample.

The monomer conversion (*α*, %) of the tested sample was calculated according to Equation (2):

(2) $$\alpha =1-\frac{Xm_{0}}{1000\times M\times (m_{1}/M_{1}+m_{2}/M_{2}+m_{3}/M_{3})}$$

where *α*—monomer conversion of tested sample; *X*—bromine number (mg g^−1^); *m*_0_, *m*_1_, *m*_2_, *m*_3_—correspond to masses of all monomers (*m*_0_ = *m*_1_ + *m*_2_ + *m*_3_), AA, AMPS, AcA, respectively; *M*, *M*_1_, *M*_2_, *M*_3_—relative molecular weights of Br, AA, AMPS, AcA.

### 2.6. Measurement of Setting Time of Cement Paste

Based on the Chinese standard JC 477-2005 [33], the initial setting time (IST) and final setting time (FST) of cement paste were determined using Vicat apparatus as follows. Initially, 400 g of cement were mixed with distilled water (144 g for an admixture dosage of 7 wt.% over cement, 140 g for 8 wt.%). This mixture was stirred at low speed for 30 s. Subsequently, the admixture (A1–A3, respectively) was added in varying quantities: 28 g for a 7 wt.% dosage, 32 g for 8 wt.%. The resulting mixture was first stirred at low speed for 5 s and then at high speed for 15 s. The prepared cement paste was immediately poured into a round mold, compacted, and lightly vibrated. The surface of the paste was then smoothed with a scraper.

Both IST and FST were recorded every 10 s using the Vicat apparatus, which involved penetrating the cement paste with a needle of fixed cross-section under constant force. The IST was measured as the time from the moment the needle was released in free fall until it reached a depth of 4 ± 1 mm from the bottom of the paste. The FST was calculated as the duration between the end of the IST and the moment when the needle could no longer penetrate the paste.

### 2.7. Measurement of Compressive and Flexural Strengths of Cement Mortar

The compressive and flexural strengths of the cement mortar were tested in accordance with the Chinese standard JC 477-2005. First, 900 g of cement was combined with distilled water (459 g for an admixture dosage of 7 wt.% over cement, 450 g for 8 wt.%) in a mixing bowl. This mixture was stirred at low speed for 30 s using a cement mortar mixer (JJ-5, made by Wuxi Xiyi Building Material Instrument Factory, Wuxi City, China). After an additional 30 s of low-speed stirring, 1350 g of Chinese ISO standard sand was gradually added.

The mixture was then mechanically stirred at high speed for 30 s, allowed to rest for 90 s, and stirred again at high speed for another 30 s. Immediately after stirring, the admixture (A1–A3) was introduced in varying amounts: 63 g for a 7 wt.% dosage, 72 g for 8 wt.%. The mixture was stirred at low speed for 5 s, followed by high-speed stirring for 15 s.

The cement mortar was then promptly transferred into a mold measuring 40 mm × 40 mm × 160 mm (trial mold for soft scouring) and stored in a cement conservation box at 20 °C with 90% humidity for predetermined incubation periods of 6 h, 24 h, and 28 d.

## 3. Results and Discussion

### 3.1. Characterization of Admixtures

**Figure 2:**
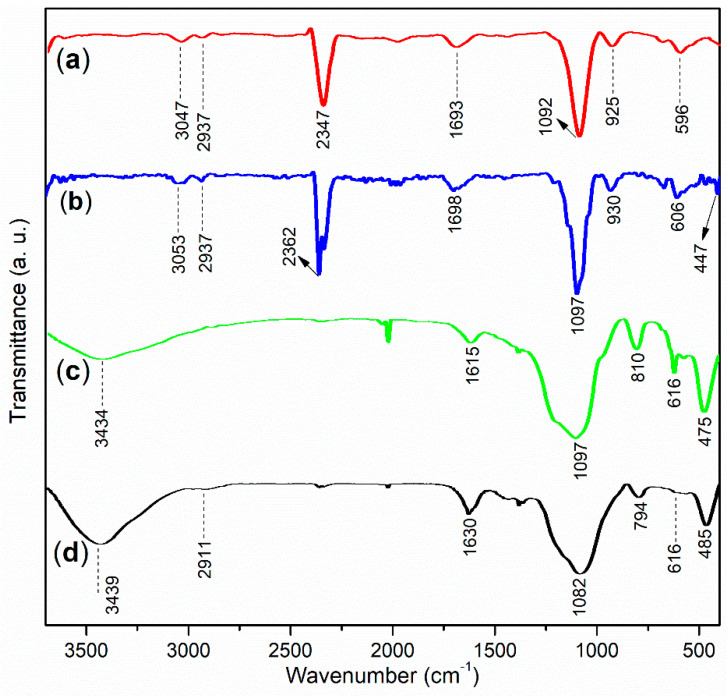
FT-IR spectra: (**a**) A1, (**b**) A2, (**c**) silica gel, (**d**) A3a.

The values of X and α for A1 were presented in Table 1. (NH_4_)_2_S_2_O_8_ appeared to be an effective initiator for the aqueous free radical polymerization of AA, AMPS, and AcA at moderate temperatures, as illustrated in Figure 1 [34]. The FT-IR spectra of the admixtures are shown in Figure 2. In Figure 2a, a moderate and broad band at 3047 cm^−1^ for A1 corresponds to the O–H stretching vibrations of carboxyl groups from AA and AcA, as well as the sulfonic group from AMPS. Additionally, the band at 2937 cm^−1^ indicates the anti-symmetric stretching of the C–H bond in the methylene groups within the A1 framework [35]. The peak at 2347 cm^−1^ was indicative of CO_2_ vibrations.

Furthermore, A1 exhibited a band at 1693 cm^−1^, likely corresponding to the C=O stretching of the carboxyl groups from AA and AcA. The subsequent peaks at 1092 cm^−1^ and 925 cm^−1^ were attributed to the anti-symmetric and symmetric stretching vibrations of the sulfonic group in AMPS, respectively.

The small band observed at 3047 cm^−1^ in A1 shifted to 3053 cm^−1^ after mixing A1 with AS to form A2 (Figure 2a,b). This shift suggested that the ordered hydrogen-bonded O–H units of the carboxyl and sulfonic groups in A1 were disrupted and coordinated with Al^3+^. Additionally, A2 displayed a weak peak at 1698 cm^−1^, which was notably higher than the 1693 cm^−1^ peak found in A1 (Figure 2a,b). This change further indicated that the hydrogen-bonded C=O units of the carboxyl groups from AA and AcA in A1 were also disrupted and coordinated with Al^3+^.

Furthermore, the peak at 1092 cm^−1^ in A1 shifted to 1097 cm^−1^ in A2 (Figure 2a,b), indicating that the S=O unit of the amide group in AMPS was coordinated with Al^3+^. Additionally, a new peak appeared at 447 cm^−1^ in A2, which was absent in A1 (Figure 2a,b). This new peak was likely due to the Al–O stretching vibration of [Al(OH)_4_]^−^ or other intermediates resulting from the hydrolysis of Al^3+^ during the preparation of the admixtures (Figure 1).

The silica gel exhibited a broad peak centered at 3434 cm^−1^ (Figure 2c), which corresponds to the vibration of SiO–H groups on the surface of the silica gel. A subsequent peak appeared at 1615 cm^−1^ (Figure 2c), primarily attributed to the stretching of the C=O bond from organic residues on the silica gel, differing from the peak observed at 1693 cm^−1^ in A1 (Figure 2a,c). Additionally, two peaks are noted at 1097 cm^−1^ and 810 cm^−1^ (Figure 2c), reflecting the anti-symmetric and symmetric stretching vibrations of the Si–O bond.

As shown in Figure 2c, when silica gel was combined with A2, the resulting A3a exhibited a larger and broader band at 3439 cm⁻¹, suggesting the vibration effects of O–H groups from silanol, carboxyl, and sulfonic groups. A smaller band at 2911 cm^−1^, attributed to the anti-symmetric stretching of the C–H bond in the methylene groups of the block copolymer framework, was also observed. Additionally, A3a displayed a moderate band centered at 1630 cm^−1^, which was lower than the 1698 cm^−1^ observed in A2 but higher than the 1615 cm^−1^ observed in the silica gel (Figure 2b–d). This shift suggested a covalent linkage between the silica gel and A2, rather than a simple mechanical mixture. The subsequent peaks at 1082 and 794 cm^−1^ reflected the vibrations of sulfonic groups and Si–O bonds, respectively. Furthermore, a peak at 485 cm^−1^ indicated the stretching of the Al–O bond in A3a.

**Figure 3:**
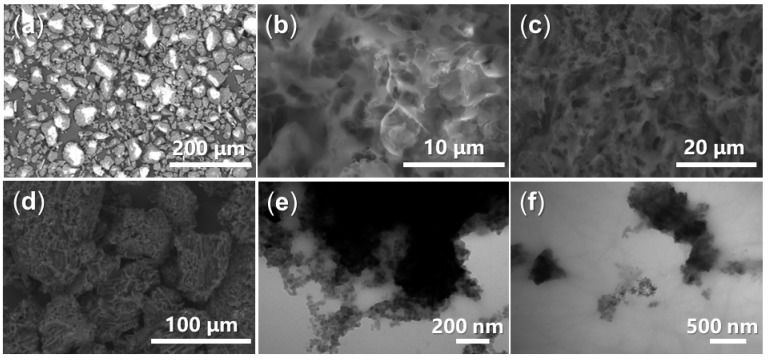
SEM images: (**a**) silica gel (magnification of 200×), (**b**) A1 (5000×), (**c**) A2 (2000×), (**d**) A3a (500×); TEM images: (**e**) A3a (100,000×), (**f**) A3a (50,000×).

On the other hand, the morphology of the synthetic intermediates and admixtures was particularly interesting. Initially, the silica gel comprised both large blocks measuring 50–60 μm and smaller particles ranging from 10–20 μm (Figure 3a). In contrast, the synthesized block copolymer (A1) appeared as large membranes (Figure 3b). Following the incorporation of AS, the membrane became agglomerated and fragmented, resulting in A2 (Figure 3b,c). When silica gel was combined with A2, it was observed that the large blocks of silica gel were enveloped by the wrinkled membranes of A2 in A3a (Figure 3d). Additionally, numerous denser particles approximately 15 nm in size were incorporated into the membranes of A3a (Figure 3e,f), suggesting the presence of Al-containing components.

### 3.2. Effects of Admixtures in Cementing

The initial setting time (IST) and final setting time (FST) of cement paste, along with the compressive and flexural strengths of cement mortar, were summarized in Table 2. Notably, in the absence of any admixtures, the IST and FST of the cement paste were significantly prolonged, and the development of compressive and flexural strengths of the mortar was slow over the periods of 6 h, 24 h, and 28 d (entry 1, Table 2).

Upon the introduction of pure AS as an admixture, both the IST and FST were notably reduced, and improvements in compressive and flexural strengths were observed at 6 h, 24 h, and 28 d (entries 2 vs. 1, Table 2). However, the resulting IST and FST (18.81 and 36.59 min, respectively, entry 2, Table 2) did not meet the requirements set forth by the Chinese standard GB/T 35159-2017 [36] (IST ≤ 5 min, FST ≤ 12 min).

The use of A1 as an admixture significantly reduced setting times and improved mechanical strengths compared to the blank experiment (entries 3 vs. 1, Table 2). This effect was likely due to the acidic nature of A1, which may inhibit the rapid hydrolysis of Al^3^⁺ ions and thereby facilitate better setting. 

In contrast, the application of A2 led to a substantial reduction in both IST and FST compared to the blank, AS, and A1 experiments, while also enhancing both the compressive and flexural strength of the mortar (entries 4 vs. 1–3, Table 2). Clearly, A2 demonstrated superior performance in accelerating setting and hardening during cement hydration.

According to previous reports, the coordination of block copolymers with Al^3+^ ions may inhibit the rapid agglomeration and hydrolysis of Al^3+^ into unreactive Al(OH)_3_ or Al_2_O_3_. This process subsequently promotes the formation of [Al(OH)_4_]^−^, which accelerates the reaction involving C_3_A (a precursor of AFt), thereby facilitating faster setting [28]. Additionally, block copolymers may act as structure-directing agents, contributing to the ordered arrangement of C_3_A or AFt on the surface of C_3_S, thereby enhancing rather than hindering the hydration of C_3_S.

When A2 was further combined with silica gel, the resulting A3a exhibited even shorter setting times compared to A2 (IST and FST, entries 5 vs. 4, Table 2). Additionally, A3a showed increased flexural strengths at 6 h, 24 h, and 28 d, while the compressive strengths at the same time intervals decreased (entries 5 vs. 4, Table 2). Previous reports have indicated that the effects of silica compounds in mortar cementing are primarily due to their ability to fill the micropores of the mortar, which clearly enhances flexural strength [26,27]. However, the newly formed phases appeared to be somewhat softer and more flexible than expected, rather than hard, which resulted in a reduction in compressive strength.

Furthermore, when the loading amount of A3a was increased from 7% to 8%, the setting times were extended, while the compressive strengths at various intervals remained largely unchanged. However, the flexural strength at 28 d slightly decreased (entries 6 vs. 5, Table 2). This observation suggests that an excessive amount of silicon dioxide may not only fill the micropores of the mortar but also lead to the formation of new, brittle phases. These phases could potentially compromise the mechanical strength of the mortar.

On the other hand, A3b exhibited significantly increased setting times, a noticeable decrease in compressive strengths at various intervals, and enhanced flexural strengths at 24 h and 28 d compared to A3a at the same dosage (entries 7 vs. 5, Table 2). This further confirms that an excessive loading of silicon dioxide can lead to the formation of new, brittle phases. While these phases may improve flexural strength to some extent, they ultimately compromise the hardness of the synthesized materials.

Lastly, A3a demonstrated a higher R28 value compared to A3b (Table 3), indicating that the incorporation of excessive silicon dioxide into the cement matrix can hinder the long-term development of the compressive strength of the mortar.

### 3.3. Experimental Insights into the Cementing Process Facilitated by Admixture

#### 3.3.1. Elemental and Composition Analysis

**Figure 4:**
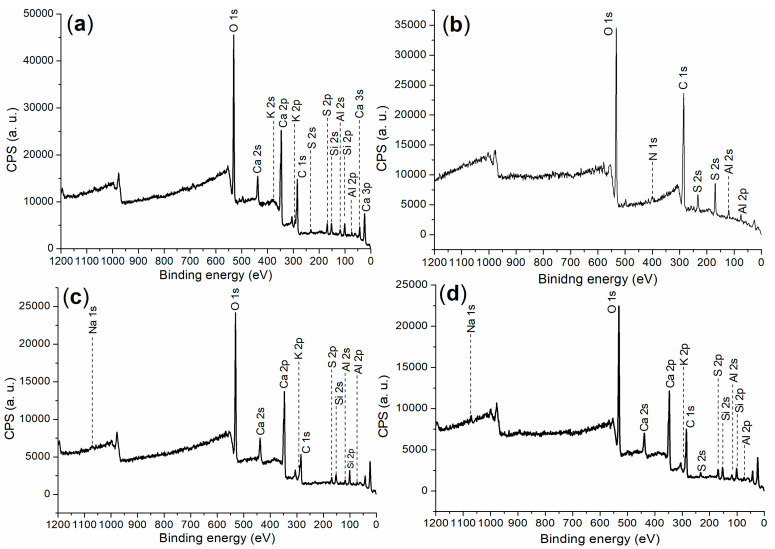
XPS survey scan: (**a**) cement; (**b**) A2; (**c**) hydrated mortar facilitated by A2, 24 h, entry 4, Table 2; (**d**) hydrated mortar facilitated by A3a, 24 h, entry 5, Table 2.

To further understand the cementing process enhanced by the admixtures, comprehensive characterizations were conducted to provide clear insights. First and foremost, Table 4 presents the composition and ignition loss of the cement, corresponding to those of typical Ordinary Portland Cement (OPC) [2]. For the cement (raw material), A2, and various hydrated mortars facilitated by different admixtures, the XPS survey scans are shown in Figure 4, while the binding energy and atomic composition are detailed in Table 5.

**Figure 5:**
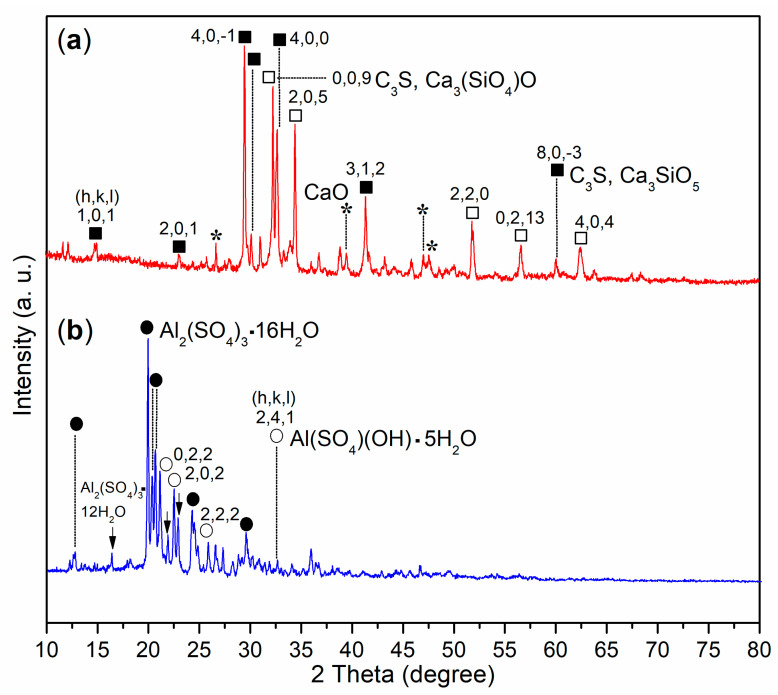
Wide-angle XRD spectra (2*θ* = 10°–80°): (**a**) cement (dark and white cubes, C_3_S; asterisks, CaO); (**b**) A2 (dark dots, Al_2_(SO_4_)_3_·16H_2_O; white circles, Al(SO_4_)(OH)·5H_2_O; arrows, Al_2_(SO_4_)_3_·12H_2_O).

First, it was interesting to examine the fine structure of the cement, which provided a foundation for the subsequent research. As illustrated in Figure 4a, the photoelectron signals for Ca, Si, and O were significantly more pronounced compared to other elements such as Al and K. The presence of C 1s photoelectron signals indicated organic residues in the cement, which correspond to the ignition loss noted in Table 4. Coupled with XRD analysis, three distinct phases were identified in the wide-angle XRD of the cement: C_3_S (dark cubes, Figure 5a; Ca_3_SiO_5_, 3CaO·SiO_2_, PDF No. 49-0442), C_3_S (white cubes, Figure 5a; Ca_3_(SiO_4_)O, 3CaO·SiO_2_, PDF No. 73-0599), and calcium oxide (asterisks, Figure 5a; CaO, PDF No. 28-0775). Clearly, the cementing process described here primarily involved the hydration of C_3_S phases.

Second, A2 presented three wide-angle XRD patterns used to characterize three new phases: Al_2_(SO_4_)_3_·16H_2_O (dark dots, Figure 5b; aluminum sulfate hydrate, PDF No. 49-1096), Al_2_(SO_4_)_3_·12H_2_O (arrows, Figure 5b; metaalunogen, PDF No. 18-0061), and Al(SO_4_)(OH)·5H_2_O (white circles, Figure 5b; rostite, PDF No. 41-1382). The former two phases propose that AS was dehydrated stepwise, while the last phase indicates that the remaining AS was extensively hydrolyzed.

**Figure 6:**
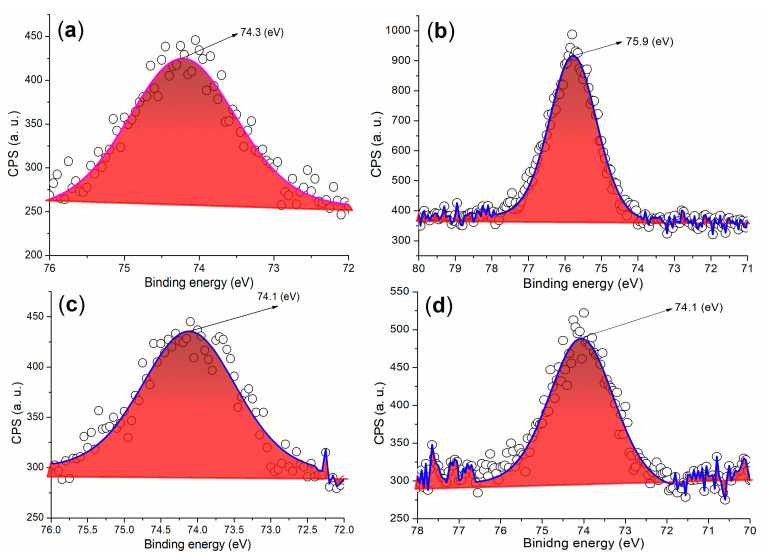
XPS measurements of the Al 2p region: (**a**) cement; (**b**) A2; (**c**) mortar of 24 h facilitated by A2, entry 4, Table 2; (**d**) mortar of 24 h facilitated by A3a, entry 5, Table 2.

To verify the results obtained from XRD, it was essential to analyze the chemical state of Al throughout the cementing process. The binding energy of the Al 2p photoelectron from the cement was centered at 74.3 eV, as shown in Figure 6a. This value was significantly higher than that of metallic Al (72.6 eV) reported previously [38], but lower than the binding energy of Al^3^⁺ in the Al_2_O_3_ phase [39]. This suggested the presence of tetrahedral Al^3^⁺ in the Al(OH)_3_ phase within the cement. In contrast, A2 exhibited a binding energy of Al 2p photoelectrons at 75.9 eV, which was notably higher than the 74.3 eV observed in cement, corresponding to the phases of Al_2_(SO_4_)_3_·16H_2_O and Al(SO_4_)(OH)·5H_2_O (Figure 5b).

**Figure 7:**
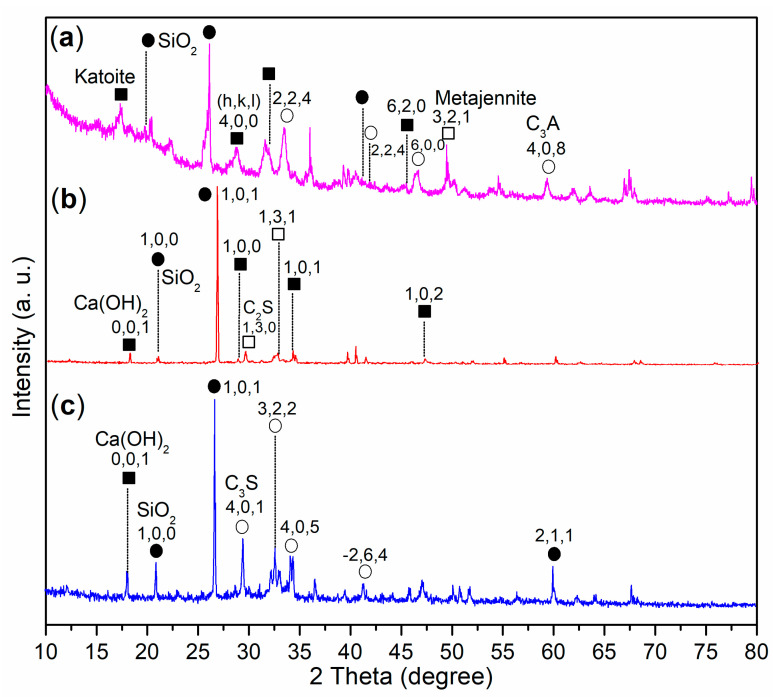
Wide-angle XRD spectra (2*θ* = 10°–80°): (**a**) mortar of 24 h facilitated by A2, entry 4, Table 2 (dark dots, SiO_2_; dark cubes, Katoite; white cubes, metajennite; white circles, C_3_A); (**b**) mortar of 24 h facilitated by A3a, entry 5, Table 2 (dark dots, SiO_2_; dark cubes, Ca(OH)_2_; white cubes, C_2_S); (**c**) mortar of 24 h, facilitated by AS, entry 2, Table 2 (dark dots, SiO_2_; dark cubes, Ca(OH)_2_; white circles, C_3_S).

Next, it was intriguing to further investigate the crystallinity of the mortar to elucidate the reasons behind the changes in mechanical strength induced by various admixtures. The XRD spectrum of the A2-facilitated mortar (24 h) revealed four diffraction systems: SiO_2_ (dark dots, Figure 7a; PDF No. 47-1144), Katoite (dark cubes, Figure 7a; Ca_3_Al_2_SiO_4_(OH)_8_, PDF No. 38-0368), metajennite (white cubes, Figure 7a; Ca_9_(Si_6_O_18_H_2_)(OH)_8_·2H_2_O, PDF No. 32-0165), and C_3_A (white circles, Figure 7a; calcium aluminum oxide, Ca_3_Al_2_O_6_, PDF No. 33-0251). 

In contrast, the pure AS-facilitated mortar (24 h) exhibited three series of diffractions, including SiO_2_ (dark dots, Figure 7c; PDF No. 65-0466), Ca(OH)_2_ (dark cubes, Figure 7c; portlandite, PDF No. 04-0733), and residual C_3_S (white circles, Figure 7c; calcium silicate, Ca_3_SiO_5_, 3CaO·SiO_2_, PDF No. 31-0301). Given that the cement primarily consisted of C_3_S (Figure 5a), it was evident that the use of A2 as an admixture significantly enhanced the hydration of C_3_S compared to pure AS.

**Figure 8:**
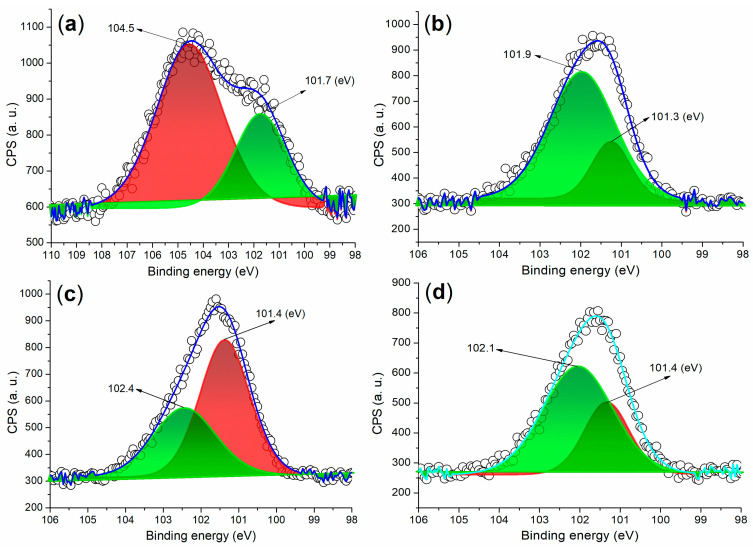
XPS measurements of the Si 2p region: (**a**) cement; (**b**) mortar of 24 h facilitated by A2, entry 4, Table 2; (**c**) mortar of 24 h facilitated by A3a, entry 5, Table 2; (**d**) mortar of 24 h facilitated by AS, entry 2, Table 2.

To further validate the results obtained from XRD, the XPS spectra of the Si 2p regions in cement and mortars facilitated by various admixtures were analyzed. The XPS spectrum of the cement revealed two fitted Si 2p peaks: the first peak at 104.5 eV, which corresponds to Si^4+^ fixed in the non-hydrated C_3_S phase, and the second peak at 101.7 eV, attributed to Si^4+^ in the amorphous SiO_2_ phase (Figure 8a) [40]. Following the hydration of mortar facilitated by A2, a new peak appeared at 101.9 eV, reflecting the contributions of Si 2p photoelectrons from Katoite and metajennite (Figure 7a and Figure 8b). Meanwhile, the other peak at 101.3 eV continued to characterize Si^4+^ in the SiO_2_ phase (Figure 8b). On the other hand, the XPS spectrum of the AS-facilitated mortar displayed two Si 2p photoelectron peaks at 102.1 eV and 101.4 eV, which were characteristic of Si^4+^ ions fixed in the C_3_S and SiO_2_ phases, respectively (Figure 8d). These findings corresponded to the phases identified in the XRD analysis (Figure 7c).

The XRD spectrum of the A3a-facilitated mortar revealed three distinct diffraction systems: SiO_2_ (indicated by dark dots, Figure 7b; PDF No. 65-0466), Ca(OH)_2_ (represented by dark cubes, Figure 7b; portlandite, PDF No. 44-1481), and residual C_2_S (denoted by white cubes, Figure 7b; calcium silicate, PDF No. 49-1672). Additionally, the XPS analysis of this mortar exhibited two Si 2p photoelectron peaks at 102.4 eV and 101.4 eV, which were both higher than those observed in the A2-facilitated mortar (Figure 8b,c). These peaks correspond to Si^4+^ ions in the C_2_S and SiO_2_ phases, respectively (Figure 7b). Compared to the AS-facilitated mortar, the A3a-facilitated mortar demonstrated a greater ability to promote the hydration of C_3_S rather than C_2_S, while pure AS could not completely transform C_3_S within 24 h (Figure 7b,c).

Lastly, from a different perspective, since A3b exhibited prolonged setting times and reduced compressive strengths but enhanced flexural strengths compared to A3a (entries 7 vs. 5, Table 2), it was of interest to analyze the elemental composition of the corresponding mortars to assess the efficiency of elemental incorporation. As presented in Table 6, the mortar from A3a had a higher silicon (Si) mass percentage but a lower aluminum (Al) mass percentage compared to that from A3b. This suggests that increasing Si content in the cement might not necessarily improve Si incorporation efficiency but could elevate Al content instead. It appears that Al-containing components may significantly influence the development of flexural strength.

#### 3.3.2. Morphological, Thermogravimetric, and Functional Group Analyses

**Figure 9:**
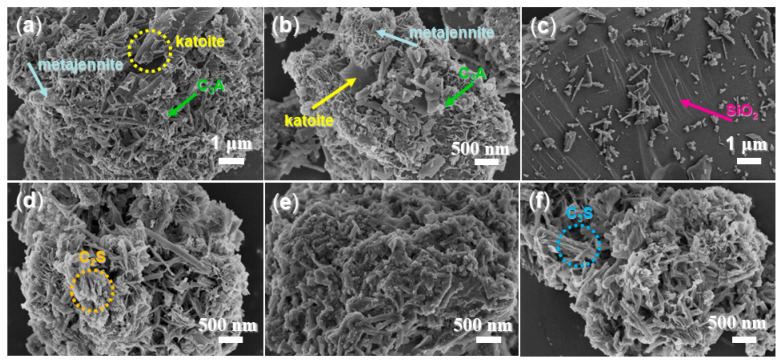
SEM images of mortar: (**a**) mortar of entry 4, Table 2 (A2-facilitated, 24 h, magnification of 10,000×); (**b**) mortar of entry 4, Table 2 (A2-facilitated, 24 h, 20,000×); (**c**) mortar of entry 4, Table 2 (A2-facilitated, 28 d, 10,000×); (**d**) mortar of entry 5, Table 2 (A3a-facilitated, 24 h, 20,000×); (**e**) mortar of entry 5, Table 2 (A3a-facilitated, 28 d, 20,000×); (**f**) mortar of entry 2, Table 2 (AS-facilitated, 24 h, 20,000×).

Initially, large, smooth flakes (indicated by yellow circles in Figure 9a and the yellow arrow in Figure 9b) were observed in the morphology of A2-facilitated mortar after 24 h. These flakes were attributed to katoite (PDF No. 38-0368, Figure 7a), with the formula Ca_3_Al_2_SiO_4_(OH)_8_. The OH^−^ in katoite may originate from Al(SO_4_)(OH)·5H_2_O in A2 (Figure 5b). Previous studies have shown that katoite served as a key binder in the hydration of calcium aluminate cement, offering resistance to aggressive environments. Additionally, katoite improved compressive strength, reduced drying shrinkage, increased bulk density, and decreased the porosity of mortar [41]. Katoite is characterized by smooth flakes approximately 1 μm × 1 μm in size [41], consistent with those observed in Figure 9a,b.

Considering that A2-facilitated mortar exhibited significantly reduced setting times, enhanced compressive strength, and greater flexibility compared to mortars with admixture blank, pure AS, and A1 (entries 4 vs. 1–3, Table 2), it was likely that the presence of Al(SO_4_)(OH)·5H_2_O as an active intermediate in A2 greatly contributed to these improvements.

Second, metajennite (Ca_9_(Si_6_O_18_H_2_)(OH)_8_·2H_2_O, PDF No. 32-0165, Figure 7a) exhibited a porous solid morphology (grey arrows in Figure 9a,b). Its formula suggests it could represent a transitional phase of C-S-H (hydrated tricalcium silicate, 3CaO·SiO_2_·3H_2_O), with OH^−^ from metajennite potentially being incorporated by Al(SO_4_)(OH)·5H_2_O from A2 as well. Previous studies have shown that excessive gypsum loading in cement can alter the C-S-H morphology from needle-like structures to globular forms within the initial hours of C_3_S hydration, significantly affecting setting times and early mechanical strength [42]. Additionally, the introduction of gypsum to cement has been found to transform the morphology from isolated needles to mesoporous clusters [42]. Therefore, it was evident that the inclusion of A2 in mortar cementing in this study influenced the formation and morphology of C_3_S, with the intermediate metajennite potentially accelerating setting times.

It was also intriguing to observe the morphology and effects of C_3_A in cementing. Previous studies have indicated that sulfate ions can inhibit the reaction of C_3_A, preventing flash setting and ultimately leading to under-sulfation [42]. However, as illustrated in Figure 9a,b, only small particles, rather than isolated needle-like structures measuring 200 nm–500 nm, were present in the mortar. This suggests that most of the C_3_A was converted when A2 was used as an admixture, rather than being retained. Furthermore, after 28 days, the formed SiO_2_ phase exhibited a smooth surface, with some residual cementing components visible (Figure 9c). This evidence supported the conclusion that the incorporation of A2 has a long-term positive effect on the mechanical strength development of the mortar (entry 4, Table 2).

The mortar facilitated by A3a (24 h) exhibited a different morphology compared to the A2-facilitated mortar (Figure 9a,b,d). XRD analysis (Figure 7b) and SEM observations (orange circle, Figure 9d) indicated the presence of only C_2_S, with detectable SiO_2_. This suggested that A3a significantly accelerated the hydration of C_3_S and C_2_A, leading to a rapid reaction without the formation of C-S-H intermediates. Consequently, the mortar with A3a showed shorter setting times, lower compressive strength, and notably higher flexural strength compared to the A2-facilitated mortar (entries 5 vs. 4, Table 4). The silica-containing components in the A3a-facilitated mortar likely filled the micropores and formed low-carbon binder in cement formulations [43]. After 28 days, the A3a-facilitated mortar exhibited increased adhesion, with no isolated needles or particles detectable on the surface. This observation indicates that the hydration process was nearly complete.

Lastly, it was important to examine the morphology of the AS-facilitated mortar. In practice, this mortar displayed only clusters of needle-like structures approximately 1 μm in length (blue circle, Figure 9f), which corresponded to the unreacted C_3_S phase identified by XRD (Figure 7c). This observation further indicated that the complexation of A1 (synthesized block copolymer) with AS was instrumental in accelerating the transformation of C_3_S, rather than the use of pure AS alone.

**Figure 10:**
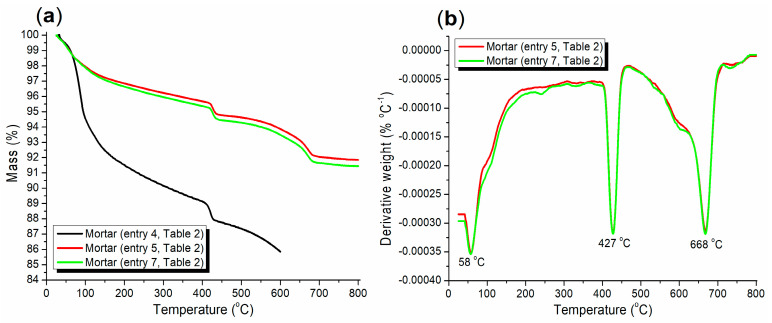
Thermogravimetric analysis for mortars (24 h, Table 2): (**a**) TGA; (**b**) DTG.

The thermogravimetric analysis, encompassing both TGA and DTG, can provide insights into the accelerating and hardening effects of the admixtures (Figure 10). Initially, the A2-facilitated mortar exhibited a steeper TGA curve compared to the mortars facilitated by A3a and A3b (black vs. red and green, Figure 10a). A weight loss of 8.49% was observed between 30 and 200 °C (black, Figure 10a), primarily corresponding to the evaporation of adsorbed or crystalline water upon heating. Subsequently, a weight loss of 4.29% occurred between 300 and 600 °C (black, Figure 10a), suggesting the release of OH^−^ from katoite (Figure 7a, Ca_3_Al_2_SiO_4_(OH)_8_, PDF No. 38-0368) and metajennite (Figure 7a, Ca_9_(Si_6_O_18_H_2_)(OH)_8_·2H_2_O, PDF No. 32-0165).

The green TGA curve consistently remained below the red one between 30 and 800 °C (Figure 10a), indicating that the incorporation of A3b as an admixture adsorbed more crystalline water or other volatile species compared to A3a, likely due to differences in silica composition during synthesis (Figure 1). However, the DTG curves for the mortars facilitated by A3a and A3b were nearly identical, with three distinct weight loss rates observed at 58 °C, 427 °C, and 668 °C. These rates can be attributed to the release of adsorbed and crystalline water, the release of OH^−^ from various salts, and changes in crystal structure, respectively.

### 3.4. Proposed Mechanism for Cement Hydration Facilitated by A2

**Figure 11:**
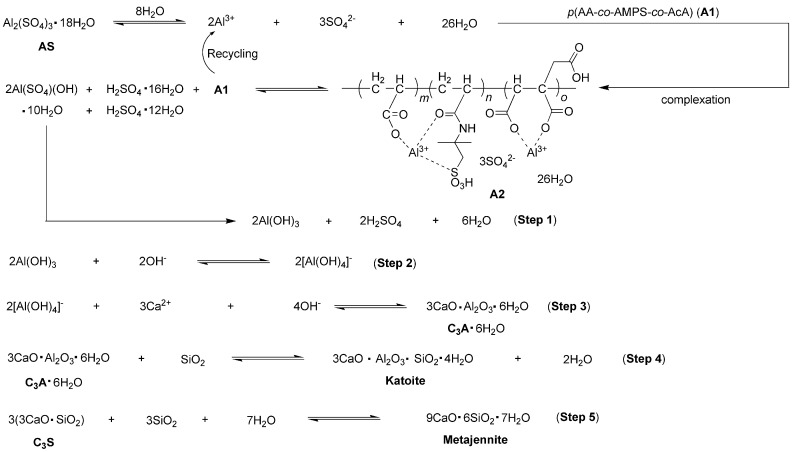
Proposed mechanism for cement hydration facilitated by A2.

Based on the results obtained thus far, the cement hydration mechanism facilitated by A2 is summarized in Figure 11. Initially, AS was ionized in water and subsequently coordinated by A1. This coordination was preferred over the direct hydrolysis of Al³⁺ into unreactive Al(OH)_3_ or Al_2_O_3_, due to the low solubility of AS (36.5 g AS per 100 g H_2_O at 20 °C) [30].

The coordination intermediate provided Al(SO_4_)(OH)·5H_2_O, which was subsequently hydrolyzed to form active Al(OH)_3_ (Step 1, Figure 11). In this process, Al(OH)_3_ was generated through the successive substitution of SO_4_^2−^ with OH^−^ from Al(SO_4_)(OH)·5H_2_O, thereby preventing the agglomeration and precipitation of Al^3+^. The resulting Al(OH)_3_ then reacted with OH^−^ to yield [Al(OH)_4_]^−^, which further interacted with Ca^2+^ to produce C_3_A (Steps 2–3, Figure 11). Then, C_3_A further reacted with SiO_2_ to form katoite (Ca_3_Al_2_SiO_4_(OH)_8_, 3CaO·Al_2_O_3_·SiO_2_·4H_2_O, Figure 7a; step 4, Figure 11). Meanwhile, the combined hydration of C_3_S with SiO_2_ provided metajennite (Ca_9_(Si_6_O_18_H_2_)(OH)_8_·2H_2_O, 9CaO·6SiO_2_·7H_2_O, Figure 7a; step 5, Figure 11). The two new obtained phases contributed to the setting times of cement paste and mechanical strengths of mortar (entries 4 vs. 1–3, Table 2).

## 4. Conclusions

In this work, a ternary water-soluble copolymer containing carboxyl, sulfonic, and amide functional groups was synthesized with high monomer conversion through ammonium persulfate-catalyzed free radical polymerization in an aqueous solution. This copolymer was subsequently complexed with aluminum sulfate and further stabilized with silica gel. Characterization revealed that the synthesized copolymer formed a large, thin membrane that effectively covered both the aluminum compounds and the silica gel blocks. Additionally, the complexation of the copolymer with aluminum sulfate resulted in the formation of Al(SO_4_)(OH)·5H_2_O.

The pure aluminum sulfate, the synthesized copolymer, the complex of the copolymer with aluminum sulfate, and two silica gel-incorporated complexes (with varying silica gel dosages) were sequentially used as admixtures in the cementing of Ordinary Portland Cement. The results are summarized as follows.

1. The admixture-blank experiment revealed significantly prolonged initial and final setting times of the cement paste, along with reduced compressive and flexural strengths of the mortar. The addition of pure aluminum sulfate and the synthesized copolymer as admixtures notably shortened the setting times and enhanced the mechanical strengths. However, these improvements still fell short of meeting the requirements specified by the Chinese standard GB/T 35159-2017.
2. The introduction of a complex admixture, combining copolymer and aluminum sulfate, not only reduced the setting times of cement paste but also enhanced the mechanical strengths of mortar compared to the use of aluminum sulfate alone. Characterization results revealed that this complex admixture led to the formation of katoite and metajennite in the mortar after 24 h, alongside C_3_A. These phases contributed to improved setting times and mechanical strength development. In contrast, the mortar treated with pure aluminum sulfate contained a significant amount of unreacted C_3_S, highlighting the superior activity of the complex admixture in accelerating cement setting.
3. The further addition of silica gel to the complex admixture would shorten the setting times of the paste even more, slightly reduce compressive strength, but enhance flexural strength compared to the initial complex admixture. The presence of residual C_2_S in the mortar treated with the silica gel-incorporated complex admixture suggests that silicon components may fill the micropores and mesopores of the mortar. This process accelerated cement setting and improved flexural strength, although it slightly decreased compressive strength.
4. Increasing the dosage of the silica gel-incorporated complex admixture negatively affected the setting times of the paste, while the compressive strength remained largely unchanged; however, the flexural strength decreased. This indicated that the cement hydration performance is highly sensitive to the amount of silicon components used.
5. Conversely, under the same dosage of admixture, the complex formulation containing a higher amount of silica gel prolonged the setting times, decreased compressive strength, but significantly improved flexural strength compared to the formulation with less silica gel.

## Figures and Tables

**Table 1:** The bromine number (*X*) and monomer conversion (*α*) of A1 ^a^.

Sample	*m* (g) ^b^	*V* (mL) ^c^	*X* (mg g^−1^) ^d^	*α* (%) ^e^
blank	-	20.66	-	-
A1	0.50	16.52	66.21	97.16

^a^ As in Section 2.5. ^b^ Mass of tested sample. ^c^ Consumed volume of Na_2_S_2_O_3_ solution in titration. ^d^ Bromine number, determined by Equation (1). ^e^ Monomer conversion, by Equation (2).

**Table 2:** Setting time of cement paste and mechanical strength of cement mortar under different admixtures ^a^.

Entry ^a^	Admixture (Dosage) ^b^	Setting Time (min, Cement Paste) ^c^		Compressive Strength (MPa, Mortar) ^d^			Flexural Strength (MPa, Mortar) ^d^		
		Initial (IST)	Final (FST)	6 h	24 h	28 d	6 h	24 h	28 d
1	blank	29.10 ± 0.71 ^e^	40.07 ± 0.85	0.7 ± 0.18	4.9 ± 0.11	22.3 ± 0.44	0.4 ± 0.02	2.8 ± 0.16	10.0 ± 0.11
2	AS (7%)	18.81 ± 0.92	36.59 ± 1.85	1.1 ± 0.19	6.1 ± 0.12	24.7 ± 0.51	0.8 ± 0.10	2.9 ± 0.11	11.6 ± 0.09
3	A1 (7%)	26.53 ± 0.16	35.69 ± 0.17	0.9 ± 0.02	6.1 ± 0.10	23.6 ± 0.31	0.4 ± 0.01	2.9 ± 0.25	11.8 ± 0.27
4	A2 (7%)	4.65 ± 0.10	8.33 ± 0.31	1.6 ± 0.03	10.4 ± 0.20	26.9 ± 0.17	0.8 ± 0.02	3.6 ± 0.15	12.9 ± 0.11
5	A3a (7%)	1.00 ± 0.02	1.95 ± 0.04	1.3 ± 0.03	10.0 ± 0.13	23.9 ± 0.05	1.3 ± 0.05	4.9 ± 0.32	13.9 ± 0.08
6	A3a (8%)	1.36 ± 0.11	2.19 ± 0.02	1.2 ± 0.01	9.9 ± 0.20	24.0 ± 0.23	1.6 ± 0.07	5.8 ± 0.26	12.7 ± 0.31
7	A3b (7%)	2.21 ± 0.17	3.07 ± 0.10	1.1 ± 0.05	7.9 ± 0.16	23.2 ± 0.49	1.5 ± 0.06	6.3 ± 0.20	16.9 ± 0.08

^a^ Experimental details as in Section 2.6 and Section 2.7. ^b^ Dosage of admixture over cement, mass percentage, as in Section 2.6 and Section 2.7. ^c^ As in Section 2.6. ^d^ As in Section 2.7. ^e^ Data: average value ± SD (standard deviation).

**Table 3:** Comprehensive strength retention ratio after 28 days.

Entry ^a^	*R*_28_ (%) ^b^
5	107
7	104

^a^ Corresponding to entries in Table 2. ^b^ Retention ratio after 28 days, *R*_28_ = *f*_t,28_/*f*_r,28_ × 100%, *f*_t,28_ means comprehensive strength of 28 d for tested mortar sample (MPa), *f*_r,28_ means comprehensive strength of 28 d for standard mortar sample (MPa, entry 1, Table 2), according to Chinese standard GB/T 35159-2017.

**Table 4:** The composition of cement. ^a^.

Composition	CaO	SiO_2_	Al_2_O_3_	Fe_2_O_3_	SO_3_	MgO	K_2_O	Na_2_O	Ignition loss ^b^
Content (wt.%)	61.06	18.02	6.01	3.76	4.55	1.69	1.25	0.33	3.33

^a^ Chemical composition of cement (raw material) was determined by ICP-OES. ^b^ Ignition loss was detected according to GB/T 34231-2017 [37].

**Table 5:** Binding energy and atomic composition of element on sample surface (depth, 0–10 nm).

Entry	C (1s)	O (1s)	S (2p)	Si (2p)	K (2p)	Ca (2p) or N (1s)	Al (2p)
Cement ^a^	284.80 (34.92) ^b^	530.80 (32.27)	168.80 (2.84)	100.80 (7.08)	292.80 (8.79)	346.80 (11.74)	73.80 (2.35)
A2	284.80 (59.72)	532.80 (28.26)	169.80 (7.31)	- ^c^	-	400.80 (1.27) ^d^	74.80 (3.44)
4 (24 h) ^e^	284.80 (30.49)	530.80 (38.53)	167.80 (1.18)	101.80 (8.67)	292.80 (7.68)	346.80 (11.70)	73.80 (1.55)
5 (24 h) ^f^	284.80 (33.02)	531.80 (35.57)	168.80 (3.11)	101.80 (6.67)	292.80 (8.32)	346.80 (10.68)	74.80 (2.07)
7 (24 h) ^g^	284.80 (33.13)	531.80 (37.05)	168.80 (3.04)	101.80 (6.28)	293.0 (8.10)	346.80 (12.15)	74.30 (0.25)

^a^ Raw material, as in Section 2.1. ^b^ Binding energy (eV), along with atomic percentage (at%) in parentheses. ^c^ Not found or not counted by instrument due to low content. ^d^ Binding energy (atomic percentage, at%) of N 1s photoelectron. ^e^ Cement mortar, corresponding to entry 4 in Table 2, in addition, Na (1s) data: 1070.80 (0.19). ^f^ Cement mortar, corresponding to entry 5 in Table 2, in addition, Na (1s) data: 1070.80 (0.56). ^g^ Cement mortar, corresponding to entry 5 in Table 2.

**Table 6:** Elemental composition of prepared mortars (24 h) ^a^.

Entry ^b^	Si (wt.%)	Al (wt.%)	S (wt.%)	Mg (wt.%)	Na (wt.%)
5	24.47	1.41	0.69	0.25	0.16
7	24.24	1.74	0.80	0.32	0.20

^a^ Elemental percentage (%) of mortar determined by ICP-OES, as in Section 2.3. ^b^ Cement mortar, corresponding to same entries in Table 2.

## Data Availability

The original contributions presented in the study are included in the article, further inquiries can be directed to the corresponding author.
